# A case-control study of tobacco smoking and tuberculosis in India

**DOI:** 10.4103/1817-1737.56007

**Published:** 2009

**Authors:** R. Prasad, Suryakant, R. Garg, S. Singhal, R. Dawar, G. G. Agarwal

**Affiliations:** *Department of Pulmonary Medicine, Chhatrapati Sahuji Maharaj Medical University (Erstwhile K. G. Medical University), Lucknow, India*; 1 *Department of Statistics, Lucknow University, Lucknow, India*

**Keywords:** Diagnosis, India, smoking, tobacco, tuberculosis

## Abstract

**OBJECTIVES:**

To evaluate the role of smoking as a risk factor for the development of pulmonary tuberculosis.

**MATERIALS AND METHODS:**

A total of 111 sputum smear—positive patients of pulmonary tuberculosis and 333 controls matched for age and sex were interviewed according to a predesigned questionnaire.

**RESULTS:**

The adjusted odd ratio of the association between tobacco smoking and pulmonary tuberculosis was 3.8 (95% confidence interval, 2.0 to 7.0; *P* value, <.0001). A positive relationship between pack years, body mass index and socioeconomic class was also observed.

**CONCLUSION:**

There is a positive association between tobacco smoking and pulmonary tuberculosis.

Nearly 17% of smokers of the world live in India.[1,2] Recent household surveys from India have shown that more than one third of men and a few percent of women who smoke tobacco are in the middle age group.[3,4] It has been consistently shown from various disease surveys in India that the prevalence of pulmonary tuberculosis among males aged ≥15 years is 2-4 times higher than in females of the same age;[5] so it might be possible that there is an association between tobacco smoking and the higher rate of tuberculosis in adolescent males.

Therefore, a case-control study was carried out to determine the association between pulmonary tuberculosis and smoking.

## Materials and Methods

This study was carried out in the Department of Pulmonary Medicine of the Chhatrapati Sahuji Maharaj Medical University. We included subjects from Uttar Pradesh only, whereas the rest were excluded. Thus a population of 166,197,921 subjects (total population of UP) was surveyed and was the source for cases and controls for the present study. Patients were recruited from September 2004 to August 2005.

Sputum smear–positive pulmonary tuberculosis patients were taken as cases. For each case, 3 controls were taken from among the healthy bystanders of that patient. They were matched for age (-5 years), sex and place. To exclude any respiratory disease, all controls were subjected to clinical evaluation, chest radiograph and sputum examination. All subjects with comorbid conditions such as diabetes mellitus, human immunodeficiency virus infection and malignancy; and those on any immunosuppressive drugs were also excluded from the study.

Informed consent was taken from all subjects. Approval for this study was also obtained from the review board of our institution. A predesigned questionnaire enquiring about smoking history, household smoke exposure, environmental smoke exposure, tobacco chewing, alcoholism, housing characteristics and score on the modified Kuppuswamy socioeconomic status scale was used as instrument for data collection. This modified Kuppuswamy socioeconomic status classification contemplates five social classes: Upper (I), upper middle (II), lower middle (III), upper lower (IV) and lower (V). Details of smoking were noted carefully with regard to type, current smoking status, age of starting smoking, duration of smoking and quantity of smoking. Trained MD students (trained in the subject of ‘tuberculosis and chest disease’) interviewed the subjects in the hospital. ‘Smoker’ was defined as a person who had smoked more than 100 cigarettes/ bidis during his/her lifetime. ‘Nonsmoker’ was defined as a person with exposure less than that stated above.

### Statistical analysis

Data was entered into Microsoft Excel and subsequently converted to an SAS file for performing univariate and multivariable analysis. Univariate analysis was carried out by computing unadjusted matched odds ratios (ORs) and their 95% CIs to compare cases and controls for each categorical variable of interest, where as *t*-test statistics was used to make the corresponding comparison for the continuous variables. Multivariable analysis was conducted through conditional logistic regression to identify risk factors independently associated with pulmonary tuberculosis and to calculate their adjusted matched odds ratios.

## Results

A total of 111 patients and 333 controls were enrolled in the study. Smoking history was present in 33.3% of the patients as compared to 13.8% of controls. The mean pack/year was 4.77 among patients and 0.88 among controls. After controlling for the effect of other variables in the model, the odds of developing pulmonary tuberculosis among smokers was 3.8 times more than that among nonsmokers [OR = 3.8 (95% CI, 2.0 to 7.0), *P* <.0001].

The risk was higher for the persons who were smoking ≥5 pack years [adjusted OR = 4.6 (95% CI, 2.1 to 10.1)] than persons who were smoking ≤5 pack years [adjusted OR = —2.9 (95% CI, 1.2 to 6.0)]. Persons smoking more number of bidis or cigarettes per day and for lesser duration and vice versa can have the same pack/year [Table 1]. Analysis was also done to answer the question, which is more hazardous, a large number of bidis or cigarettes per day or a long duration of smoking [Table 1]? The odds of developing pulmonary tuberculosis among persons who smoked ≤10 bidis or cigarettes per day [adjusted OR = 4.0 (95% CI, 1.7 to 9.1)] was slightly more in comparison to persons who smoked ≥10 bidis or cigarettes per day [adjusted OR = 3.6 (95% CI, 2.4 to 13.1)]. However, the odds of developing pulmonary tuberculosis among persons who smoked for a duration of ≥10 years [adjusted OR = 5.7 (95% CI, 2.4 to 13.1)] was more in comparison to persons who smoked for a duration of ≤10 years [adjusted OR = 2.5 (95% CI, 1.1 to 5.7)]. These analyses reveal that long duration of smoking is more hazardous than a large number of bidis or cigarettes per day.

**Table 1:** Association of pulmonary tuberculosis with smoking

Variables	Cases 111 (100%)	Controls 333 (100%)	Matched OR (95% CI)	Adjusted* OR (95% CI)	*P* value
Pack year#					
≤5	15 (13.5)	27 (8.1)	2.4 (1.1, 5.0)	2.9 (1.2, 6.8)	0.02
≥5	22 (19.8)	19 (5.7)	4.6 (2.3, 9.2)	4.6 (2.1, 10.1)	0.0001
No. of bidis or cigarettes per day#				
≤10	19 (17.1)	24 (7.2)	3.4 (1.7, 7.0)	4.0 (1.7, 9.1)	0.001
≥10	18 (16.2)	22 (6.6)	3.4 (1.7, 6.8)	3.6 (1.7, 7.9)	0.001
Duration of smoking (years)#				
≤10	15 (13.5)	30 (9.0)	2.0 (1.0, 4.2)	2.5 (1.1, 5.7)	0.03
≥10	22 (19.8)	16 (4.8)	5.6 (2.7, 11.8)	5.7 (2.4, 13.1)	<0.0001

# Reference category is ‘nonsmoker.’;

* Adjusted for BMI, social class and house type

Analyses were also done to assess the association between pulmonary tuberculosis and various factors like socioeconomic status, body mass index (BMI), type of housing, alcohol intake and environmental exposure [Tables 2 and 3]. The odds of developing pulmonary tuberculosis among social class V [adjusted OR = 5.3 (95% CI, 1.8 to 16.0)] was more than that among social class IV [adjusted OR = 2.3 (95% CI, 0.8 to 6.7)] with reference to social class type III. Alcohol intake was also found to have an association with the occurrence of pulmonary tuberculosis [adjusted OR = 1.7 (95% CI, 0.9 to 3.3)]. Having BMI < the median value of 19.4 was strongly associated with pulmonary tuberculosis [adjusted OR = 4.1 (95% CI, 2.5 to 6.8)]. Persons living in kuchcha or semi-pucca houses [adjusted OR = 3.2 (95% CI, 1.4 to 7.5)] had almost similar odds of developing pulmonary tuberculosis when compared with persons living in pucca houses [adjusted OR = 2.3 (95% CI, 1.0 to 5.1). Other factors like chewing tobacco, alcohol, mosquito coil and biomass fuel were not found to be associated with pulmonary tuberculosis in the univariate analysis [Table 3].

**Table 2:** Multivariable logistic regression model for the factors associated with pulmonary tuberculosis

Variables	Matched OR (95% CI)	Adjusted OR (95% CI)	*P* value
Smoking	3.4 (2.0, 5.8)	3.8 (2.0, 7.0)	< 0.0001
Social class*			
Type V	5.3 (1.8, 16.0)	3.6 (1.0, 12.8)	0.04
Type IV	2.3 (0.8, 6.7)	1.9 (0.6, 6.4)	0.3
House type**			
Kuchcha	3.2 (1.4, 7.5)	2.8 (1.1, 7.2)	0.03
Semi-*pucca*	2.3 (1.0, 5.1)	2.3 (0.9, 5.6)	0.07
Body mass index***	4.1 (2.5, 6.8)	4.2 (2.4, 7.3)	< 0.0001

* Reference category is ‘type III socioeconomic status.’;

** Reference category is ‘pakka house.’;

*** Reference category is ‘BMI > 19.4 (median value)

**Table 3:** Univariate analysis of various other factors for their possible association with pulmonary tuberculosis

Variables	Cases 111 (100.0%)	Controls 333 (100.0%)	Matched OR (95% CI)
Chewing tobacco	24 (21.6)	76 (22.8)	0.9 (0.6, 1.6)
Alcohol	19 (17.1)	37 (11.1)	1.7 (0.9, 3.3)
Mosquito coil	25 (22.5)	70 (21.0)	1.1 (0.7, 1.9)
Biomass fuel	29 (26.1)	92 (27.6)	0.9 (0.6, 1.5)

## Discussion

In our case-control study of 111 sputum smear–positive pulmonary tuberculosis (TB) patients and 333 controls, the odds of developing pulmonary tuberculosis among smokers was 3.4 times more than that among nonsmokers, a figure that increased to 3.8 after controlling for the effect of other confounders like socioeconomic status, body mass index and house type [OR = 3.8 (95% CI, 2.0 to 7.0)]. Most of our subjects belonged to low socioeconomic class, in which bidi smoking is the prevalent mode of smoking. The relatively low combustibility and nonporous nature of the tendu leaves (used in manufacturing of bidis) require more frequent and deeper puffs by the smoker to keep bidis lit, which is therefore more harmful to active smokers as compared to passive smokers.

The above result is in agreement with the previous Indian studies, which also report the higher odds ratio of developing tuberculosis among smokers as compared to nonsmokers.[6–9] Recent meta-analysis[10] also showed that OR for TB disease ranged from 2.33 (95% CI, 1.97 to 2.75) to 2.66 (95% CI, 2.15 to 3.28). The reason for the increased risk of developing pulmonary tuberculosis among smokers is not clear, but the increased risk among smokers may be explained by the effects of smoking on pulmonary host defenses. Chronic exposure to tobacco, as well as to a number of environmental pollutants, impairs the normal clearance of secretions on the tracheobronchial mucosal surface and may thus allow the causative organism, Mycobacterium tuberculosis, to escape the first level of host defenses, which prevent bacilli from reaching the alveoli.[11] Smoke also impairs the function of pulmonary alveolar macrophages (AMs), which are not only the cellular target of *M. tuberculosis* infection but also constitute an important early defense mechanism against the bacteria; AMs isolated from the lungs of smokers have reduced phagocytic ability and a lower level of secreted proinflammatory cytokines than do those from the lungs of nonsmokers.[12] Recent work has suggested a novel mechanism for the effect: Nicotine is hypothesized to act directly on nicotine acetylcholine receptors on macrophages to decrease production of intracellular tumor necrosis factor and thus impair killing of M. tuberculosis.[13] These effects of smoking on pulmonary host defense support a causal link between smoke exposure and either an increased risk of acquiring TB or progression of TB to a clinical disease. In our study, duration of smoking was found to be more significantly associated with development of pulmonary tuberculosis in comparison to quantity of bidis or cigarettes. According to other studies, stronger association was found to be present between numbers of bidis or cigarettes per day and development of pulmonary tuberculosis.[6,7] This difference may be due to the difference in sample size. In our study, socioeconomic status, alcohol and BMI were also found to have significant association with the development of tuberculosis, whereas the type of house in which one lived and non-inhalation mode of tobacco exposure were not found to be significantly associated. This is in conformity with other studies.[14,15] Recent meta-analysis found substantial evidence that passive smoking and indoor air pollution increased the risk of TB disease.[16] It can be concluded that smoking is associated with high prevalence of tuberculosis in India. Therefore, in India, where both smoking and tuberculosis are common conditions, preventing initiation of smoking and promoting quitting of smoking are important TB-preventive measures.

### Limitation

Most of the patients attending our hospital are of low and middle socioeconomic class. Therefore, our sample may not be the true representative of the population.
